# Factors Associated with Medication Nonadherence among Hypertensives in Ghana and Nigeria

**DOI:** 10.1155/2015/205716

**Published:** 2015-10-05

**Authors:** Vincent Boima, Adebowale Dele Ademola, Aina Olufemi Odusola, Francis Agyekum, Chibuike Eze Nwafor, Helen Cole, Babatunde L. Salako, Gbenga Ogedegbe, Bamidele O. Tayo

**Affiliations:** ^1^School of Medicine and Dentistry, University of Ghana, P.O. Box 4236, Accra, Ghana; ^2^Department of Medicine, Korle Bu Teaching Hospital, P.O. Box 77, Accra, Ghana; ^3^Department of Paediatrics, Faculty of Clinical Sciences, College of Medicine, University of Ibadan, Ibadan PMB 5017, Oyo State, Nigeria; ^4^Department of Paediatrics, University College Hospital, Ibadan PMB 5116, Oyo State, Nigeria; ^5^Department of Medicine, General Hospital, Randle Avenue, Apapa PMB 1014, Lagos, Nigeria; ^6^Cardiology Division, Department of Medicine, University of Port Harcourt Teaching Hospital, Port Harcourt PMB 6173, Rivers State, Nigeria; ^7^Department of Population Health, NYU School of Medicine, 227 E. 30th Street, 6th Floor, New York, NY 10016, USA; ^8^Department of Medicine, Faculty of Clinical Sciences, College of Medicine, University of Ibadan, Ibadan PMB 5017, Oyo State, Nigeria; ^9^Department of Medicine, University College Hospital, Ibadan PMB 5116, Oyo State, Nigeria; ^10^Global Institute of Public Health, New York University, New York, NY 10003, USA; ^11^Department of Public Health Sciences, Stritch School of Medicine, Loyola University Chicago, 2160 South First Avenue, Maywood, IL 60153, USA

## Abstract

*Background.* Blood pressure (BP) control is poor among hypertensives in many parts of sub-Saharan Africa. A potentially modifiable factor for control of BP is medication nonadherence (MNA); our study therefore aimed to determine factors associated with MNA among hypertensives in Ghana and Nigeria.* Methodology.* We conducted a multicenter cross-sectional study. Patients were recruited from Korle-Bu Hospital (*n* = 120), Ghana; and University of Port Harcourt Teaching Hospital, (*n* = 73) Apapa General Hospital Lagos (*n* = 79) and University College Hospital Ibadan (*n* = 85), Nigeria.* Results.* 357 hypertensive patients (42.6% males) participated. MNA was found in 66.7%. Adherence showed correlation with depression (*r* = −0.208, *P* < 0.001), concern about medications (*r* = −0.0347, *P* = 0.002), and knowledge of hypertension (*r* = 0.14, *P* = 0.006). MNA was associated with formal education (*P* = 0.001) and use of herbal preparation (*P* = 0.014). MNA was found in 61.7% of uninsured participants versus 73.1% of insured participants (*P* = 0.032). Poor BP control was observed in 69.7% and there was significant association between MNA and poor BP control (*P* = 0.006). *Conclusion*. MNA is high among hypertensives in Ghana and Nigeria and is associated with depression, concern about hypertensive medications, formal education, and use of herbal preparations. The negative association between health insurance and MNA suggests interplay of other factors and needs further investigation.

## 1. Introduction

Hypertension is a common but treatable public health problem globally. It is estimated to cause 7.5 million deaths annually, about 12.8% of all deaths worldwide [1]. Globally, the prevalence of hypertension in adults was approximately 40% in 2008 [2]. The number of people worldwide with hypertension rose from 600 million in 1980 to nearly 1 billion in 2008. The burden of hypertension is particularly high in sub-Saharan African countries. The reported prevalence of hypertension in Ghana ranges from 19% to 32.8% in rural areas and 25.5 to 48% in urban areas [3–5]. Similarly, recent studies in Nigeria showed that the prevalence of hypertension in rural areas ranges from 21 to 25% [6, 7] while in semiurban and urban areas the prevalence ranged from 27 to 46% [8–11]. Optimal blood pressure (BP) control with levels below 140/90 mmHg is associated with significant reduction in cardiovascular complications such as stroke and coronary heart disease [12, 13].

The increasing rates of hypertension in sub-Saharan Africa have been attributed to rapid epidemiologic transition from an agrarian lifestyle to a more westernized lifestyle, with increasing rates of obesity, unhealthy diet, and physical inactivity [3, 14]. Blood pressure control is generally poor among hypertensive patients in sub-Saharan Africa, and efforts to improve BP control are needed. Poor BP control among hypertensives in sub-Saharan Africa is related to the complex interplay of patient, provider, and socioeconomic factors in this region [3, 14]. For example, patients may lack knowledge about hypertension, or they may harbour beliefs that are discordant with those of the traditional medical model regarding the causes and treatment of hypertension. As a result, patients' beliefs may be discordant with practices associated with BP control potentially leading to poor medication adherence. Additionally, patients may exhibit poor medication adherence because they are unable to afford the cost of medications. Similarly, healthcare providers may have insufficient time and resources to provide the necessary education and treatment.

Of the patient factors that affect BP control, poor medication adherence is the most salient and little studied in sub-Saharan African countries including Nigeria and Ghana. Medication nonadherence (MNA) is a potentially modifiable risk factor for the improvement of BP control. Factors that may influence adherence include socioeconomic status, access to health insurance, depression, patient's knowledge of the disease, and beliefs about medications [15]. Past studies have reported a wide range of MNA in Ghana and Nigeria from 17.4 to 93%, making it difficult to compare the degree and factors associated with MNA in both countries [16–24].

In order to address these significant gaps in the adherence literature in sub-Saharan Africa, our study documents the levels and factors associated with MNA among hypertensive patients in the hospital setting in Ghana and Nigeria.

## 2. Methods

In this multicenter cross-sectional study, patients were recruited from four hospitals: Korle-Bu Hospital (*n* = 120), Ghana, and University of Port Harcourt Teaching Hospital, (*n* = 73) Apapa General Hospital Lagos (*n* = 79), and University College Hospital Ibadan (*n* = 85), Nigeria. Patients were eligible to be in this study if they were aged 18 years or older, were diagnosed hypertensive, and had been placed on medication for at least twelve months and provided informed consent. Hypertension was defined as systolic BP of ≥140 mmHg and/or diastolic BP of ≥90 mmHg [25] or patients already under treatment with antihypertensive medications. Patients were recruited from general outpatient or specialist medical clinics. The questionnaires were administered by the authors or trained research assistants. At each site, each consecutive patient who met the inclusion criteria was recruited. The same recruitment procedure was used in each center. Patient recruitment into the study took place between April and September 2013. Data collection was conducted by the authors and trained research assistants at each study site using a structured questionnaire. Blood pressure was measured with an Omron electronic BP machine, after at least 10 minutes rest, in the dominant arm of seated patients on three occasions at an interval of one minute. The average of the last 2 readings was recorded.

The dependent variables were medication adherence and BP control. Adherence to antihypertensive medications was measured using the 8-item Morisky medication adherence scale. Based on a pilot study in Korle Bu Ghana, the response options for the Morisky scale were modified from “never/rarely,” “once in a while,” “sometimes,” “usually,” and “all the time” to “Never/rarely” versus “sometimes/usually/all the time.” Individual item scores were summed and those with a score of 8 were considered adherent to medication. Medium adherence corresponded to a score of 6–8, while a score of <6 was considered low adherence [26]. Medication nonadherence referred to a score of <8 (i.e., medium or low adherence). Blood pressure control was defined as systolic BP < 140 mmHg and diastolic BP < 90 mmHg [27].

Demographic and socioeconomic characteristics including age, gender, ethnicity, education, occupation, and income were recorded. Educational status was classified as none, primary, junior secondary, senior secondary, and tertiary. Monthly income was stratified as <$100, $101–300, $301–1000, $1001–3000, and >$3000. Clinical information regarding when the diagnosis of hypertension was made, for how long patients had been on antihypertensives, and presence of any associated comorbid conditions were obtained from patients' medical records. Beliefs about medication were measured using the modified beliefs about medication questionnaire (BMQ) [28]. The BMQ includes questions on beliefs about the necessity of taking medications (BMQ-necessity) and concerns about medications (BMQ-concern). The difference between the BMQ-necessity and BMQ-concern scores was also determined for each patient (BMQ necessity-concern score). Each question on the BMQ is based on a 5-point Likert scale with response options: “strongly agree,” “agree,” “disagree,” “strongly disagree,” and “uncertain.” Pilot testing of the BMQ indicated that participants were more comfortable with 3 response options. We therefore modified the responses to “agree,” “disagree,” and “uncertain,” The parameters were scored as disagree = 1, uncertain = 2, and agree = 3. The sum total of the BMQ-necessity score, BMQ-concern, and the difference between BMQ-necessity and concern scores was calculated [28]. The Patient Health Questionnaire 9 (PHQ-9) was used to assess depression in this study. For this scale, sores of 0, 1, 2, and 3 are assigned to response categories of “not at all,” “several days,” “more than half the days,” and “nearly every day,” respectively. The PHQ-9 total score ranges from 0 to 27. PHQ-9 scores of 0–4, 5–9, 10–14, 15–19, and 20–27 represent none to minimal, mild, moderate, moderately severe, and severe depression, respectively. Scores of >4 were classified as depression. A 17-item questionnaire based on the JNC 7 and adapted from previous studies was used to assess knowledge of hypertension [14, 29–31]. The questions were based on true or false responses to statements such as “high blood pressure cannot be cured but can be controlled,” “high blood pressure can damage hearts,” “a person who has high blood pressure should take more salt,” and “management of high blood pressure must include medications, diet, and lifestyle modification.” A score of 1 mark was given for each correct response and the final score was a sum total of the correct responses.

Data collected was entered into Statistical Package for Social Sciences (SPSS) software student version 17.0 and used for analysis. Descriptive statistics such as means, standard deviations, proportions, and percentages were used to summarize quantitative and qualitative variables, respectively. Inferential statistics including Chi-square analysis and Pearson's correlation were used to compute associations between variables. The student *t*-test was used to compare means. *P* values < 0.05 were considered significant and *r*-values of >0.15 were considered strong correlations. Ethical approval for the study was obtained from University of Ghana Ethical and Protocol Review Committee (EPRC), the University of Ibadan/University College Hospital Ibadan Ethical Review Committee, and the University of Port Harcourt Ethical Review Committee.

## 3. Results

As shown in Table 1, a total of 357 hypertensive patients were recruited for the study, of which 42.6% were men. Their mean age was 56.6 ± 13.2 years. The majority of the study participants (33.61%) were recruited from Korle Bu, while 20.4%, 22.1%, and 23.8% participants were recruited from Port Harcourt, Apapa, and Ibadan, respectively. There were significant differences in the age of participants, gender, and access to health insurance between sites. The cohort from Korle Bu had the highest proportion of females and participants who were more likely to have health insurance.

Nonadherence to medications was present in 66.7% of participants. Port Harcourt had the highest prevalence of MNA (95.9%), while Ibadan had the lowest (45.1%). Depression was present in 31.4% of the study participants, with 2.8% and 1.4% reporting moderately severe and severe depression, respectively. Depression was more prevalent in the cohort from Korle Bu (41.7%) and Port Harcourt (41.1%) compared to the other centres. Table 1 shows the distribution of patient characteristics by site.

### 3.1. Relationship among Depression, Beliefs about Medicines, and Medication Adherence

The mean age of patients who were nonadherent to medications was 54.5 ± 13.2 years while those who were adherent had a mean age of 60.9 ± 12.1 years (*P* < 0.001). Among the cohort from Korle Bu, those who were nonadherent were also significantly younger than subjects who were adherent, 55.3 ± 13.4 years versus 61.3 ± 13.7 years (*P* = 0.037). There was significant correlation between PHQ-9 score for depression and Morisky score in Korle Bu (*r* = −0.230, *P* = 0.012) and in the pooled data (*r* = −0.203, *P* < 0.001). The mean BMQ-concern score among participants who were nonadherent was 9.0 ± 2.7 compared to 7.5 ± 2.5 among those who were adherent. There was a significant negative correlation between BMQ-concern score and Morisky score in Ibadan (*r* = −0.338, *P* = 0.002) and in the pooled data (*r* = −0.355, *P* = 0.000). The BMQ-necessity-concern score was associated with MNA in the pooled data (*r* = 0.336, *P* < 0.001). Knowledge of hypertension was significantly but weakly correlated with Morisky score (*P* = 0.006, *r* = 0.14).  Table 2 shows correlations among Morisky score and depression, beliefs about medication, knowledge of hypertension, and income by site.

### 3.2. Relationship among Socioeconomic Status, Income, Health Insurance, and Medication Adherence

Level of income did not show correlation with Morisky score (*r* = 0.021, *P* = 0.744). The majority of participants (56.3%) did not have any health insurance. MNA occurred in 47.7% of those who did not receive formal education compared with 70.9% of those who had some form of formal education (*P* = 0.001). MNA was noted in 61.7% of those who did not have health insurance and in 73.1% of those who had health insurance (*P* = 0.032). MNA was significantly more common among patients who used herbal preparation for the treatment of systemic hypertension (*P* = 0.014). There were no differences in medication adherence between patients who were married compared with those who were not. See Table 3 for a summary of characteristics by adherence.

### 3.3. Correlations of Blood Pressure Control

Blood pressure was controlled in 35.9% of study participants. There was variation in the proportion of patients with BP control across the study sites ranging from 9.6%, in Port Harcourt, to 54.1% in Ibadan. There was significant association between medication nonadherence and poor BP control in the pooled data (*P* = 0.006). Table 1 includes rates of BP control and medication adherence across sites. The cohort of patients from Port Harcourt had the highest level of MNA (95.9%) and the lowest proportion with BP control (9.6%, *n* = 4).

## 4. Discussion

We report a high prevalence of MNA (66.7%) among hypertensives in hospitals in Ghana and Nigeria. Patients who were nonadherent were significantly younger than patients who were adherent. There was significant negative correlation between depression and concerns about medication and medication nonadherence. Knowledge of hypertension was positively correlated with adherence. Patients who were adherent were less likely to use herbal preparations or to have received formal education and were more likely to have children. Level of income was not associated with adherence. Not unexpectedly, poor BP control was significantly associated with MNA. Access to health insurance surprisingly showed significant association with MNA. The proportion of patients who had uncontrolled BP was higher among patients who had health insurance, compared to patients without health insurance, but the finding was not significant. Further studies on the operation of the health insurance system are warranted while measures to improve medication adherence are required.

We report a high prevalence of MNA in our study population (66.7%), but our finding is within the range of MNA that has been documented in previous studies from sub-Saharan Africa. Most studies in Nigeria and Ghana have documented MNA ranging from 32.7 to 49.3% [16, 17, 20, 23]. There was however a study from Ghana that documented MNA of 93% before the availability of National Health Insurance [19]. Elsewhere in sub-Saharan Africa, MNA ranged from 10.3 to 87.5% [32–38]. Variation may be partly due to the different definitions or methods used to assess MNA. Most of the studies though based on self-report, utilized different definitions for MNA. An advantage of our study was that the same tool was used to assess MNA in the various sites. Furthermore we also observed significant association between MNA and BP control in our study which will further validate the tool that was adopted to assess MNA in our study.

Our study found significant correlation between beliefs about medication and adherence to antihypertensives. Patients who had concerns about antihypertensive medications were less likely to be adherent to their medications. These findings are consistent with previous studies on the relationship between medication adherence and beliefs about medications [39]. Specifically, patients in our study had concerns about potential adverse effects of long term use of antihypertensives or felt that daily use of antihypertensive medications was a significant disruption of their lifestyles. Patients were also concerned that they may be becoming too dependent on their medications. Other studies have identified side-effects of antihypertensive medications as a reason for MNA in patients [19, 23, 35, 38]. Studies have also documented that forgetting to take medications is an important barrier to adherence [17, 23, 38]. In addition, adherence is usually better with medications that require less frequent administration [16, 36–38]. Health education specifically about antihypertensive medications including potential side-effects, and inquiry about side-effects the patient may be experiencing and use of medications that are associated with fewer side effects may lead to improvement in adherence. In addition, use of medications that do not cause more disruption of the patients' daily activities than necessary may also improve adherence.

Our study also found that depression was significantly associated with MNA, particularly among patients at Korle Bu, but also in the pooled data. To the best of our knowledge this study is the first to assess depression and MNA among hypertensives in sub-Saharan Africa. Depression has been associated with poor BP control and medication non-adherence in studies outside sub-Saharan Africa [40, 41]. In a study from the USA, antidepressants were associated with significantly longer initial persistence of antihypertensive therapy among patients with depression who developed hypertension [42]. Evaluation for depression and appropriate management of the depression may improve medication adherence among hypertensives in sub-Saharan Africa.

Patients who were not adherent were significantly younger than patients who were adherent. This pattern has been observed in adherence studies carried outside sub-Saharan Africa and has also been documented in Sub-Saharan Africa [26, 43, 44]. The reason for worse medication non adherence in younger patients is not clear. A potential reason may be that younger patients are not as concerned about their health compared with the older patients. Health education directed towards younger age group may improve outcomes among the younger patients.

Our study found positive and significant correlation between knowledge of hypertension and adherence to medications. Previous studies from Africa have associated lack of knowledge regarding the curability of hypertension and life-long need for antihypertensives with MNA [18, 20, 23, 38]. Our data thus support the need for adequate health education on hypertension to improve MNA in the subregion. Furthermore, patients who reported using herbal preparations for the treatment of hypertension were more likely to show MNA. This is consistent with most studies on hypertension from sub-Saharan Africa that have associated the use of alternative medical therapy with MNA [17, 18, 20, 36, 38].

Studies from Nigeria have noted increasing adherence with increasing educational status but these associations were not significant [16, 23]. Literacy has also been associated with a higher BP control in Nigeria but this was also not significant [45]. Our study, however, found that patients who had received any form of formal education were more likely to show MNA than those who did not. It may be that educated participants in our study were more skeptical towards the use of antihypertensives. A similar observation was noted in a Ghanaian study which also found significant negative association between educational status and medication adherence [17]. Another study from the United States found higher adherence among men with lower levels of education than among more highly educated men [46]. Our data is consistent with a previous suggestion that health education on hypertension and its treatment should be provided for all hypertensive patients in our subregion irrespective of the patients' educational status [47].

In most studies from sub-Saharan Africa participants have identified inability to afford the cost of medications as an important barrier to medication adherence [17–19, 33, 36, 38]. A positive relationship between cost and MNA has also been found in studies outside of Africa [39] Studies have however not consistently demonstrated association of medication adherence with level of income. Our study did not find an association between MNA and income [23, 39, 43]. One explanation may be that in many African studies, as in ours, only a negligible number belong to the high socioeconomic class, making it difficult to access MNA in the high socioeconomic class [23, 45]. On the other hand the lack of association between income and MNA may reflect an interplay of other factors that contribute to medication adherence. A study carried out in Nigeria in a setting in which antihypertensives were offered for free, noted that BP control was low though higher than in settings where medications were not given for free suggesting that MNA may not be entirely due to cost [48].

We found that access to National Health Insurance was significantly associated with MNA. In addition, BP tended to be higher in patients with access to health insurance than those who did not. Most of the patients who had access to National health Insurance were from Ghana. The Ghanaian patients also had the highest proportion of patients with depression and the depression may have contributed to the high proportion of patients with MNA in the Ghanaian patients in spite of the access to health Insurance. In addition, the way the health insurance system operates may also be contributory. In Ghana for instance, a three-month prescription from clinicians is usually dispensed in three tranches where patients are given a one month supply and asked to come back for refill of their medications for the other two months. Some patients do not return for refill of their medication because of lack of travel funds or lack of a system to remind them to go for the refill. This argues in favour of a critical review of the current national health insurance system in Ghana. Esunge and colleagues in Cameroon had suggested that factors that improved MNA among hypertensives in low resource settings were free medications, free hospital visits, free transportation, open discussion with medical staff, use of common dialects, and politeness of medical staff [49].

Another method that may contribute to improvement in medication adherence in low resource settings is the use of phone technology. Use of text messages has been associated with improvement in adherence to medications [50]. Use of phone technology reminders even in low resource settings may be useful in improving medication adherence in a large number of patients.

Strengths of our study include the multicenter nature of the study, and the use of uniform tools to assess medication non-adherence, depression, knowledge of hypertension, and beliefs about medication across the sites. Limitations of our study are that the study is hospital-based and most patients were from tertiary health centers. As such, the patients in our study are likely to have more comorbidities and to be more highly motivated than patients in the community. Therefore, proportion of patients who are non-adherent in the community may be higher than the observation in our study. Furthermore, MNA was assessed by verbal report and this may also potentially underestimate adherence because of recall bias or social desirability bias, and this may also apply to the other self-report measures. A more objective measure such as urine antihypertensive drug assay, may have demonstrated a higher degree of MNA. However, the study provides valuable information on the burden of MNA among hypertensives and the potential influence of factors such as beliefs about medications, depression, and health insurance on MNA.

## 5. Conclusion

We studied MNA among hypertensives in hospitals in Ghana and Nigeria. MNA was found in 66.7% of participants. MNA occurred in younger patients and in patients who had varying degrees of depression or were concerned about their medications. Knowledge of hypertension was positively and significantly correlated with adherence. MNA was associated with use of herbal preparations, and formal education. Expectedly adherence was significantly associated with BP control. We also found that MNA was associated with health insurance, and this may be related to either underlying depression or the mode of operation of the health insurance. The finding of significant association between health insurance and MNA underscores the need for studies to identify the underlying causes of this association. Treatment of depression in patients with hypertension and depression may improve outcomes. Patients irrespective of educational status need education concerning treatment of hypertension and side-effects of medication in addition to education on hypertension to improve MNA. Other methods to improve medication adherence such as use of phone technology should also be studied.

## Figures and Tables

**Table 1:** Distribution of study population characteristics by site.

Parameter	Korle Bu	P. Harcourt	Lagos	Ibadan	Pooled

Total (%)	120 (33.6)	73 (20.4)	79 (22.1)	85 (23.8)	357 (100)
Age (years)^a^	57.0 ± 13.7	47.4 ± 12.5	57.8 ± 9.8	62.9 ± 11.5	56.6 ± 13.2
Females (%)^a^	70 (58.3)	37 (50.7)	56 (70.9)	42 (49.4)	205 (57.5)
Insurance *N* (%)^a^	114 (95)	20 (27.4)	5 (6.3)	17 (20)	156 (43.7)
HTN with comorbidities (%)^a^	57.4	20.5	34.2	62.4	45.8
Depression (%)					
No (%)	70 (58.3)	43 (58.9)	60 (75.9)	72 (84.7)	245 (68.6)
Mild (%)	32 (26.7)	18 (24.7)	18 (22.8)	10 (11.8)	78 (21.8)
Moderate (%)	10 (8.3)	5 (6.8)	1 (1.3)	3 (3.5)	19 (5.3)
Mord-severe (%)	4 (3.3)	6 (8.2)	—	—	10 (2.8)
Severe (%)	4 (3.3)	1 (1.4)	—	—	5 (1.4)
Total (%)	**120 (100) **	**73 (100) **	**79 (100) **	**85 (100) **	**357 (100) **
BMQ-necessity [median (IQR)]	14 (11–15)	11 (9–13)	15 (13–15)	10.5 (7–13)	
BMQ-concern [median (IQR)]	7.5 (5–11)	11 (9–12.5)	7 (8-9)	7 (5–9)	
Knowledge about HTN [median (IQR)]	15 (14–16)	13 (11–14)	13 (12–15)	14 (13–15)	14 (13–15)
Nonadherence (%)	72.5	95.9	53.2	45.9	66.7
Medium adherence (%)	41.7	12.3	38.0	32.9	32.8
Low adherence (%)	30.8	83.6	15.2	12.9	33.9
Blood pressure control (%)^a^	27.7	9.6	53.2	54.1	35.9
Educational status					
None (%)	6.7	1.4	53.2	16.5	18.2
Primary (%)	7.5	31.5	19.0	21.2	18.2
Junior secondary (%)	31.7	12.3	8.9	1.2	15.4
Senior Secondary (%)	15.0	28.8	13.9	18.8	18.5
Tertiary (%)	39.2	26.0	5.1	42.4	29.7
Occupation^a^					
Petty trader (%)	24.6	12.3	45.5	13.1	23.9
JCS (%)	23.7	19.2	6.5	3.6	14.2
SCS (%)	11.0	15.1	2.6	14.3	10.8
Businessman (%)	11.0	20.5	22.1	17.9	17.0
Unemployed (%)	4.2	5.5	15.6	1.2	6.3
Housewife (%)	0.8	9.6	0	1.2	2.6
Others (%)	1.7	9.6	2.6	0	3.1
Artisans (%)	5.9	0.0	1.3	7.1	4.0
Retired (%)	16.9	8.2	3.9	41.7	18.2
Use of herbal preparation (%)^a^	15.8	35.6	11.7	9.4	17.5

^a^ *P* < 0.005.

BMQ: beliefs about medication questionnaire; IQR: interquartile range; HTN: hypertension; JCS: junior civil servant; P. Harcourt: Port Harcourt; SCS: senior civil servant.

**Table 2:** Correlation of medication adherence (Morisky score) with depression, beliefs about medication, knowledge of hypertension, or income by site and in the pooled data.

Parameter	Korle Bu		Port Harcourt		Lagos		Ibadan		Pooled	
	*r*	*P*	*r*	*P*	*r*	*P*	*r*	*P*	*r*	*P*

Depression	−0.230	0.012	0.051	0.667	−0.043	0.705	−0.035	0.749	−0.203	0.000
(PHQ-9)										
BMQ-necessity	0.142	0.124	−0.079	0.507	0.091	0.433	0.002	0.984	−0.087	0.103
BMQ-concern	−0.148	0.111	−0.286	0.014	−0.014	0.905	−0.336	0.002	−0.355	0.000
BMQ-necessity-concern	0.215	0.020	0.174	0.141	0.079	0.505	0.210	0.060	0.337	0.000
Hypertension knowledge scale	0.024	0.797	0.212	0.071	0.019	0.865	−0.030	0.783	0.144	0.006
Income	0.139	0.143	−0.057	0.634	−0.200	0.081	0.099	0.419	−0.067	0.226

BMQ-concern: beliefs about medication concern score; BMQ-necessity: beliefs about medication necessity score; BMQ-necessity-concern: beliefs about medication necessity-concern score; *P* = *P* value; *r* = Pearson's correlation coefficient.

**Table 3:** Distribution of study population characteristics by adherence (*N* = 357).

Population Characteristics	Nonadherent	Adherent	*P* value
	*N* (%)	*N* (%)	

Gender			0.623
Male	104 (68.4)	48 (31.6)	
Female	134 (65.4)	71 (34.6)	
Study Site			<0.001
Korle-Bu	87 (72.5)	33 (27.5)	
Port Harcourt	70 (95.9)	3 (4.1)	
Lagos	42 (53.2)	37 (46.8)	
Ibadan	39 (45.9)	46 (54.1)	
Age	54.4 ± 13.2 years	60.9 ± 12.1 years	0.000
Educational status			0.006
None	31 (47.7)	34 (52.3)	
Primary	48 (73.8)	17 (26.2)	
Junior	42 (76.4)	13 (23.6)	
Secondary	47 (71.2)	19 (28.8)	
Tertiary	70 (60.6)	36 (34.0)	
Formal education			0.001
Yes	207 (70.9)	85 (29.1)	
No	31 (47.7)	34 (52.3)	
Marital status			0.967
Married	172 (66.9)	85 (33.1)	
Not married	66 (66)	34 (34)	
Children			0.04
Yes	217 (65.2)	116 (34.8)	
No	21 (87.5)	3 (12.5)	
Occupation			0.317
Employed	176 (68.5)	81 (38.5)	
Unemployed	59 (62.1)	36 (37.9)	
Cost of drug ($)			0.1
<10	58 (73.4)	21 (26.6)	
11–30	87 (65.9)	45 (34.1)	
31–50	36 (67.9)	17 (32.1)	
51–100	27 (69.2)	12 (30.8)	
>100	14 (45.2)	17 (50.8)	
Insurance			0.032
Yes	114 (73.1)	42 (26.9)	
No	124 (61.7)	77 (38.3)	
Use of herbal medication			0.014
Herbal med.	50 (80.6)	12 (19.4)	
No herbal med.	186 (63.5)	107 (36.5)	
Hypertension			0.006
Control	72 (56.7)	55 (43.3)	
Uncontrolled	163 (71.8)	64 (21.2)	
Comorbidities			0.874
No comorbidity	132 (69.8)	57 (30.2)	
DM or renal comorbidities	62 (62.6)	37 (37.4)	
Other comorbidities	38 (69.8)	23 (30.2)	

DM: diabetes mellitus.
